# Development and Evaluation of a Train-the-Trainer Workshop for Hong Kong Community Social Service Agency Staff

**DOI:** 10.3389/fpubh.2017.00015

**Published:** 2017-02-13

**Authors:** Qianling Zhou, Sunita M. Stewart, Alice Wan, Charles Sai-cheong Leung, Agnes Y. Lai, Tai Hing Lam, Sophia Siu-chee Chan

**Affiliations:** ^1^School of Public Health, The University of Hong Kong, Hong Kong, China; ^2^Department of Psychiatry, University of Texas Southwestern, Medical Center at Dallas, Dallas, TX, USA; ^3^Information Systems and Technology Branch, Social Welfare Department, Hong Kong, China; ^4^School of Nursing, The University of Hong Kong, Hong Kong, China

**Keywords:** train-the-trainer, capacity building, training evaluation, community-based intervention, social service agency staff, positive psychology, social work

## Abstract

**Introduction:**

Capacity building approaches are useful in large-scale community-based health promotion interventions. However, models to guide and evaluate capacity building among social service agency staff in community settings are rare in the literature. This paper describes the development and evaluation of a 1-day (7 h) train-the-trainer (TTT) workshop for the “Enhancing Family Well-Being Project”. The workshop aimed at equipping staff from different community agencies with the knowledge and skills to design, implement, and evaluate positive psychology-based interventions for their clients in Sham Shui Po, an over-crowded and low-income district in Hong Kong.

**Methods:**

The current TTT extended and improved on our previous successful model by adding research and evaluation methods (including the Logic Model, process evaluation, and randomized controlled trial), which are important to plan and evaluate the community interventions. Evaluation of the TTT was guided by the Integrated Model of Training Evaluation and Effectiveness (IMTEE), with quantitative and qualitative methods. Quantitative data were collected from pretraining (T1), post-training (T2), and 6-month (T3) and 12-month (T4) follow-up surveys. Qualitative data were collected from four focus groups of agency staff after the intervention.

**Results:**

Ninety-three staff from 30 community agencies attended the training, and 90 completed the baseline survey. Eighty-eight, 63, and 57 staff performed the evaluations at T2, T3, and T4, respectively. Agency staff were satisfied with the TTT. Immediate enhancement of knowledge, self-efficacy, and positive attitudes toward the training content was found at T2 (Cohen’s *d* ranged from 0.24 to 1.22, all *p* < 0.05). Enhancement of knowledge of all training contents persisted at T3 and T4 (Cohen’s *d* ranged from 0.34 to 0.63, all *p* < 0.05). Enhancement of self-efficacy in the use of positive psychology in intervention design persisted at T3 (Cohen’s *d* = 0.22, *p* = 0.04). The skills learned were utilized to plan and develop subsequent interventions. Twenty-nine interventions were successfully designed and implemented by the agency staff, and delivered to 1,586 participants. The agency staff indicated their intention to utilize the skills they had learned for other interventions (score ≥4 out of 6) and to share these skills with their colleagues. Qualitative feedbacks from 23 agency staff supported the quantitative results.

**Conclusion:**

Our brief TTT was effectively delivered to a large number of agency staff and showed effects that persisted up to 12 months. Our training and evaluation models may offer a template for capacity building among social service agency staff for community brief, universal family health promotion interventions in diverse settings.

## Introduction

This paper describes a short train-the-trainer (TTT) workshop and its evaluation, in a community-based intervention project designed to improve family well-being in Hong Kong, under the “FAMILY: a Jockey Club Initiative for a Harmonious Society” (“The FAMILY Project”, http://www.family.org.hk) (1). The documentation of the impact of training programs for delivery of interventions is scare (2). The TTT presented here was guided by an evaluation model (3) and directly evaluated for its effectiveness. The TTT was a 1-day (7 h) workshop to build community social service agency staff’s capacity for the intervention and the science of evaluation. Agency staff were expected to immediately use the knowledge and skills acquired to design, implement, and evaluate positive psychology-based interventions for participants recruited from their communities. In the TTT framework, experts train the interventionists to deliver services (4). This strategy enables low cost, preventive, and population-wide health promotion interventions (5). The academic and community collaborative approach (6) that drives the TTT has been shown to work well outside the West (7), but there are few studies that include rigorous evaluation of TTTs.

Traditional primacy of family in Chinese life is under threat in Hong Kong, the most urbanized and westernized city in China (1, 8–11). High level of family well-being may serve as important protective factors for vulnerable subgroups. The FAMILY Project, which is funded by the Hong Kong Jockey Club Charities Trust, was launched to promote family health, happiness, and harmony (3Hs). The TTT reported here was run specifically for the Enhancing Family Well-Being (EFWB) Project, one in a series of community-based interventions that were part of the FAMILY project. These interventions were collaboratively developed and implemented by the Schools of Public Health and Nursing, the University of Hong Kong, and government and many non-governmental organizations (NGOs) across Hong Kong, with the aim to promote family well-being in the region. The EFWB Project was run in collaboration with the Sham Shui Po District Social Welfare Office of the government Social Welfare Department and 30 district-based NGOs. Sham Shui Po District, located in Kowloon area of Hong Kong, is one of the most densely populated districts in Hong Kong (12). The proportion of vulnerable individuals such as single parents, elderly, new immigrants from mainland China, ethnic minorities, and comprehensive social security assistance recipients in this district are relatively high, whereas the median monthly domestic household income is the lowest among all districts in Hong Kong (13). There are 226 social service units representing about 43 NGOs in the district (14).

The literature suggests that the TTT approach has been broadly used for capacity building in community health settings. For example, it has been implemented to train personnel who serve victims with domestic violence (15) and ethnic minorities with health disparities (16). It has been adopted for training in perinatal depression screening (17), parenting support (18), and education on mental health and aging (19). TTTs have also been utilized to increase social service agency staff’s interests in dissemination and implementation research in health (20). However, the application of TTT is mainly reported in the Western settings. Information about how the TTTs are developed outside the West, and adequate and systematic evaluation of their effectiveness are rarely reported in the literature. The TTT approach was used in the FAMILY Project, to enhance the competence and performance of social service staff from the participating community agencies, in designing, implementing, and evaluating the subsequent community-based interventions.

The TTT approach follows the public health principles to deliver cost-effective interventions that are likely to be sustained. Utilization of staff on the ground trained by experts has been widely used in community health settings. The TTT approach can be used to accommodate concerns that have been expressed about the dissemination of evidence-based practice and sustainability of interventions developed and implemented by academics. First, many psychosocial interventions have been developed in traditional, academic laboratory contexts. The interventions after the study is completed are rarely adopted and delivered in community settings (21), and some face the challenge of maintaining program fidelity in applied settings (22). The TTT builds capacity of the agency staff, so that they can equitably engage in every aspect of the research and program process (23). Cultural acceptability and relevance of the interventions can therefore be enhanced, and ongoing and future academics-community collaborations can therefore be promoted. Second, many concepts behind developing programs and testing the effectiveness of new programs are not understood or seen to be feasible by most community practitioners (24, 25). The TTT promotes capacity building by passing on some simple skills and tools that are beneficial to developing and testing interventions but are new to community practitioners. Agency staff’s involvement can lower the burden of human resource for implementation and evaluation of our large-scale interventions. Third, building the capacity of a large number of agency staff from different agencies could contribute to sustainable changes as a result of skill transfer to many organizations. In addition, evaluation results of the TTT are important to drive improvement in future training and help to explain the impact of the interventions (26). The inclusion of TTT evaluation results is a key contribution of the present paper.

The TTT approach has been successfully used in the Happy Family Kitchen (HFK) Project, the first in the series of the community-based interventions under the FAMILY project. The TTT of the HFK project was a 2-day workshop (12 h) developed and implemented in partnership with community agencies in the Yuen Long district. The workshop was delivered to 50 agency staff and was reported to successfully enhance agency staff’s competence and performance in applying positive psychology constructs in their family interventions. Following the workshop, the staff developed and implemented 23 interventions (7).

The current TTT of the EFWB Project was based on the public health principles stated above and used experience from our previous projects. The training program was designed to teach agency staff intervention and evaluation skills, which were essential for the subsequent interventions they delivered. This paper aims to present the development and evaluation of the current TTT. The specific objectives of our TTT were to enhance agency staff’s knowledge and acceptance of, and self-efficacy in using positive psychology, the Logic Model, process evaluation, and the RCT design in the EFWB Project and to promote the application of knowledge and skills taught.

## Materials and Methods

Following the principles underlying the TTT (i.e., to deliver cost-effective and sustainable interventions), the current training extended the TTT of the HFK project with modifications and improvements, in an attempt to enhance agency staff’s competence to conduct community-based intervention and evaluation. The current TTT was hosted in the University of Hong Kong on 21 February 2012.

### Samples

All the major NGOs working in the Sham Shui Po district were invited to a briefing session about the EFWB project, organized by the Sham Shui Po District Social Welfare Office and the academic partners from the Schools of Public Health and Nursing in December 2011. Thirty NGOs participated and submitted a brief proposal of their programs to the research team before the TTT. Agency staff from these organizations were assigned by their supervisors to participate in the TTT (free of charge) and were given feedback to improve and finalize their proposal to be completed within 2 weeks after the training. Financial support from the FAMILY project to run the programs was given to each participating NGO once its proposal was approved. The community-based interventions started about 1–5 months after the TTT. Most of the organizations had service agencies in this district serving particular client groups, such as low-income groups and new immigrants.

### Development of the Training Program

**Figure 1:**
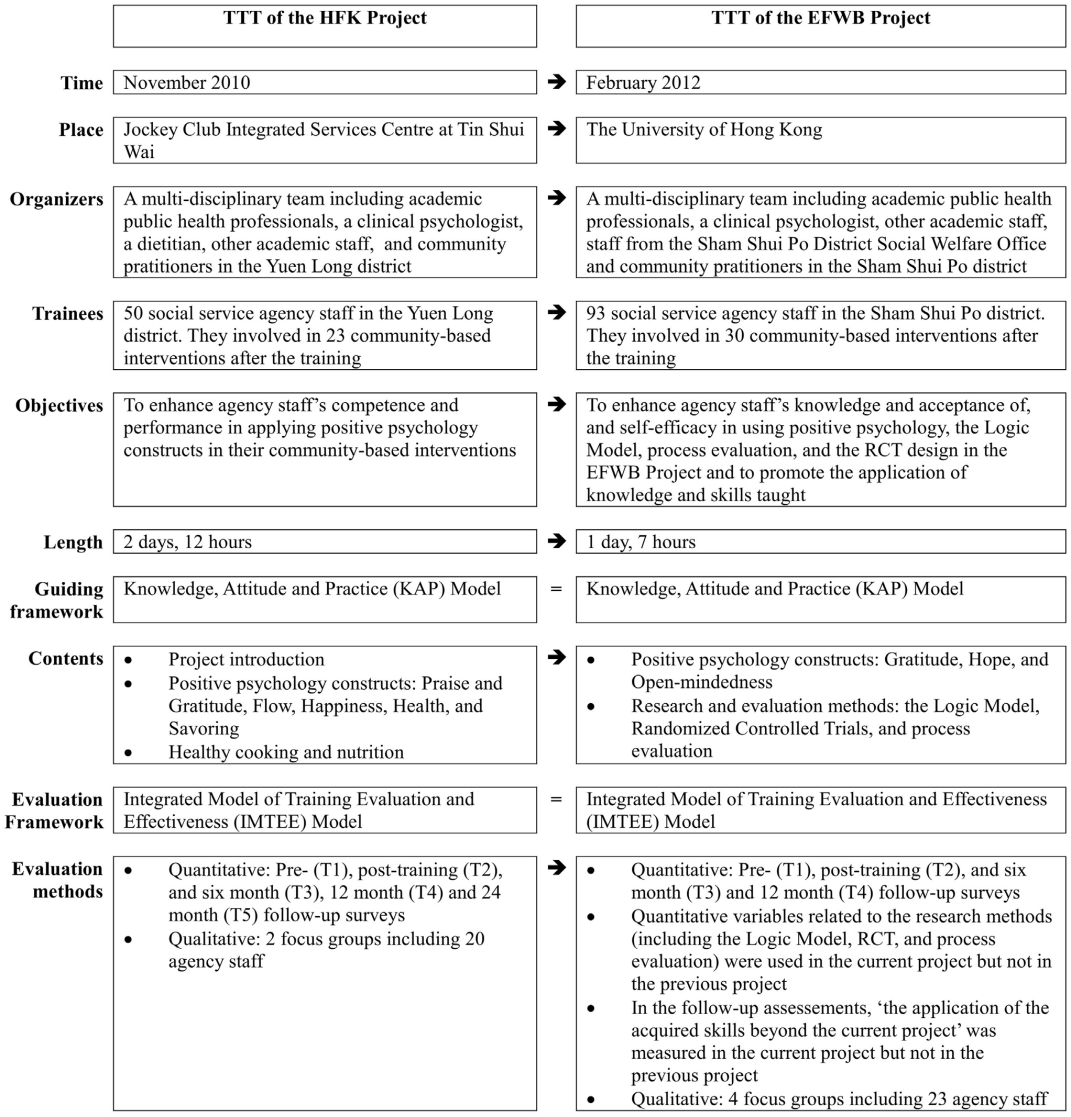
**Train-the-trainer (TTT) workshop and its evaluation: from the Happy Family Kitchen Project to the Enhancing Family Well-Being (EFWB) Project**.

Figure 1 shows how our TTT was changed from the TTT of the HFK Project. First, the length of the training was reduced from 2 days (12 h) to 1 day (7 h), by eliminating contents on healthy cooking and nutrition that were not the focus of the EFWB Project. There is evidence that brief trainings are effective and cost effective (27–29). Brief training limits the burden on the participants and enhances acceptability. Second, the current TTT was disseminated to a larger number of community agency staff, which could have greater sustainability and community impact. Third, the current TTT gave a brief introduction of the general concept and its application to research and evaluation methods, including the Logic Model (30, 31), randomized controlled trials (RCTs), and process evaluation (32), which had not been emphasized and evaluated in previous training (see below for more detail). It helped push the boundaries of community practitioners’ skill sets, as their background and training was oriented toward service delivery rather than theory and research. Understanding the research and evaluation methods was needed to plan, deliver, and evaluate the community intervention program, such as the EFWB Project that involved various community agencies and diverse service targets working under a large project with the same similar theoretical base and outcomes, and required a certain degree of flexibility of the interventions among different agencies for different participants. Fourth, the current evaluation extended the Integrated Model of Training Evaluation and Effectiveness (IMTEE) framework (3) and included more quantitative variables (e.g., the application of the acquired skills beyond the current project) to reflect transfer performance. This evaluative information indicates persistence of effects and is a key to improving the scope and reach of the training. Finally, the evaluation of the current TTT included more focus groups than the previous TTT, and groups were formed by agency staff in the same arm, in order to strengthen the qualitative evaluation.

### Contents of the Training Program

The training content was designed by a multidisciplinary team including academics and community partners basing on the “Knowledge, Attitude and Practice (KAP)” model (33) and was organized into two sessions (3 and 4 h per session) delivered on the same day. The academics partners took the lead for theoretical and research design components of the training, and the community partners ensured the acceptability of the training contents and led the component of positive psychology training.

Session I was delivered by a clinical psychologist with experience in teaching positive psychology in the social service sector. This session taught the general concept, applications, and theme-related targeted behaviors of three specific positive psychology themes designated by the EFWB Project, including “Gratitude” (34), “Hope” (35), or “Open-mindedness” (34). The main purpose was to introduce theme-related experiential activities as an example of promoting targeted behaviors in the interventions. Each community intervention implemented by the agency staff was required to be based on one of these three themes. Given different groups of clients served and potential participants to be recruited by the agencies, flexibility of choice of theme allowed the autonomy to choose the theme that was most appropriate. Details on theme selection and theme-related targeted behaviors have been described elsewhere (36).

Session II was delivered by academics in public health (Author THL) and nursing (Author SC) who were key investigators in the FAMILY Project. This session focused on the general concepts of program design, implementation, and evaluation. First, we introduced the Logic Model with a pictorial description. The Logic Model is as an operational plan that uses narrative or graphical depictions to illustrate a sequence of cause-and-effect relationships, i.e., how specific inputs and activities are linked and will accomplish intended short-, intermediate-, and long-term outcomes (30, 31). The application of the Logic Model allows precise communication about the purpose of a project, the components of a project, and the sequence of activities and accomplishments. In the current TTT, we taught agency staff key concepts of the Logic Model and provided a practical example of its application in program design. With the application of the Logic Model, we intended that all activities and interventions from the different NGOs were linked with the same objectives, core messages, and outcomes (i.e., increase in attitude, intention and practice of targeted behaviors devised from positive psychology, and improved family well-being).

Second, we explained the rationale and principle of implementing RCT, and the responsibilities of the interventionists in an RCT, in an attempt to enhance agency staff’s acceptance of the RCT design of our EFWB Project and its limitations. An RCT is a type of scientific experiment, which aims to reduce confounding and bias when testing a new intervention, and the result is classified as Level I evidence (37). However, community practitioners’ and clients’ acceptance of RCT has been found to be low because no-treatment control groups are not seen as a valid utilization of resources but appear to be deceptive to clients in community-based interventions (38, 39). In the EFWB Project, as individual randomization was not feasible, a two-arm cluster-RCT was used to assess the effectiveness of the intervention program plus a theory-based booklet (Arm A) compared to the intervention program without the booklet (Arm B).

Last, we introduced the concept of process evaluation and its application, so as to teach agency staff to evaluate the process of their own intervention programs. Process evaluation is an assessment of program process, to understand when, where, how, and in which context the intervention/program was implemented, and to what extent the intervention/program was implemented as planned (32). Process evaluation included six domains: fidelity (the extent to which intervention was implemented as planned), dose delivered (the amount of intended units of each intervention delivered by interventionists), dose received (the extent of engagement of participants in the program and the participants’ satisfaction with program), reach (the proportion of the intended priority audience that participated in the intervention), recruitment (the procedures used to approach and attract participants), and context (the environmental issues that might affect the intervention implementation or study outcomes) (32). Process evaluation is an important supplement to outcome evaluation as it elucidates reasons for success or failure of the intervention and informs replication and improvement of the intervention. The implementation of process evaluation is increasing (32). Some programs have successfully involved community practitioners in the process evaluation (40). Given the large number of interventions that were delivered in our EFWB Project, there was an important economic advantage to train the agency staff to conduct process evaluations on their own programs. This strategy lowered the overall costs of the project by reducing dependence on outside evaluators.

Based on the principles of adult learning (41, 42), diversified learning methods were used, including didactic instruction, games, role plays, and group discussion (Table 1). We prepared a graphic diagram for practicing the application of the Logic Model, written guidelines for the logistic arrangement and program implementation, and checklists for process and outcome evaluation. We also provided post-training support for the agency staff for designing, drafting the proposal for funding, and implementation (for example, program promotion materials, souvenirs, and workbooks for the participants), and a hotline for consultation and seeking technical assistance, if needed.

**Table 1:** **The curriculum of the train-the-trainer workshop**.

Session	Topic	Conducted person	Objectives	Methods and experiential activities
I	Theme specific positive psychology (3 h)	A clinical psychologist	To introduce specific positive psychology themes: “Gratitude” “Hope” and “Open-mindedness.” To introduce theme-related experiential activities as an example of promoting targeted behaviors in the interventions.	Didactic presentation to introduce the general concept (1.5 h). A theme-related puzzle game to enhance the understanding of each theme and its targeted behaviors (30 min). A role-play to enhance self-efficacy in conducting theme-related activities (1 h).
II-A	The Logic Model (1.5 h)	A professor of nursing	To introduce the key concepts of the Logic Model and its components. To show a practical example of applying the Logic Model in program design.	Didactic presentation to introduce the general concept (40 min). Group exercise to use the Logic Model graphic diagram in program design (30 min). Group discussion to exchange opinions and explore barriers to the application of the Logic Model (20 min).
II-B	Randomized controlled trial (1 h)	A professor of public health	To introduce the general concepts of RCT and the rationale of its application. To explain the role and duties of the interventionists in implementing an RCT.	Didactic presentation to introduce the general concept (45 min). Questions and answers session to further explain the rationale and application of an RCT (15 min).
II-C	Process evaluation (1.5 h)	A professor of nursing	To introduce the six domains of process evaluation and its application. To illustrate the importance of each domain of program evaluation.	Didactic presentation to introduce the general concept (50 min). Group exercise to use a checklist for program evaluation (20 min). Group discussion to exchange opinions and explore barriers of using the checklist (20 min).

### Data Collection

The surveys for TTT evaluation were designed by the academic partner and discussed with the multidisciplinary team to ensure that the components of the evaluation model be appropriately included. The baseline survey (T1) was pilot tested on five agency staff in the multidisciplinary research team for ease of understanding and redundancy. The community partners in the research team were responsible for data collection. Agency staff who attended the training session were asked to complete the self-administered questionnaire before (T1) and immediately after (T2) the training. Participants were asked to complete the follow-up questionnaire 6 months (T3) and 12 months (T4) after the training. Participants who did not complete T3 or T4 were reminded by at least two emails or phone calls. The main reasons for loss to follow-up at T3 or T4 were participants had changed jobs, they were too busy at work, or they were on vacation at time of assessments.

Semi-structured focus groups with agency staff who participated in the EFWB projects were arranged by the community partner, upon the completion of the community-based intervention (December 2012). Each NGO was asked to recruit one to two agency staff for the qualitative component of the study. Homogenous segmentation was applied to control the group composition. Groups were formed according to their RCT arm (control vs intervention) and positive psychology theme (gratitude vs hope/open-mindedness), with five to seven agency staff in each group. The academic partner designed the interview guide and conducted the interview, with one moderator and two note-taking observers in the interview panel. Each focus group opened with a short session to explain the purpose of the study and assure the participants of the confidentiality of the discussion. Focus groups were conducted in Cantonese, audiotape-recorded, in a community agency center, and lasted about 1 h. Tapes were transcribed verbatim after the interviews. Qualitative data were generated from both transcripts and notes.

Ethical approval for the study was obtained from the Institutional Review Board of the University of Hong Kong/Hospital Authority Hong Kong West Cluster. Participants in the surveys and focus groups were assured that all information would be kept anonymous and confidential and was gathered for research purposes only. Written consent was sought from each participant before the assessments.

### Measures

The IMTEE combines variables of training evaluation and effectiveness for comprehensive assessment (3) and was designed to evaluate training in the business world. Guided by the IMTEE, our TTT was evaluated on three dimensions, including reactions to the training content and design, changes in the learner, and organization payoffs.

#### Quantitative Measures

*Reactions to the training content and design* were measured by questions on participants’ satisfaction with the training content, design, and arrangement, and participants’ ratings of the overall quality, practicability, and satisfaction of the training. These questions were included at T2, with a 5-point Likert scale. Higher scores indicated greater satisfaction.

*Changes in the learner* were shown by the changes in variables across four evaluation time points (T1, T2, T3, and T4). The variables included knowledge of (cognitive learning), self-efficacy to use, and attitudes toward training contents, including positive psychology, the Logic Model, and process evaluation. Since enhancing agency staff’s skills in designing an RCT was beyond the goal of the current TTT, only variables on knowledge and attitudes, but not self-efficacy to design RCT were included in the evaluation. Participants responded to each item on a 6-point Likert Scale. Higher scores indicated more or greater knowledge, self-efficacy, intention, and positive attitude.

*Organization payoffs* were assessed by transfer performance, as measured at T3 and T4 by (a) the application of the learning (including positive psychology, the Logic Model, and process evaluation, and encouraging participants to use positive psychology strategies to improve family relationships and 3Hs) within and beyond the EFWB Project, assessed by a 5-point Likert scale from never (0) to very frequently (5); (b) participants’ intention to apply the learning to other projects (6-point Likert scale); and (c) participants’ willingness to teach the skills learned to their colleagues (“yes” and “no”). Since positive psychology had been promoted in the Sham Shui Po district by the social service sectors about 18 months prior to the EFWB Project (36), some agency staff could have had exposure to and experience in using positive psychology before the TTT. Information on the application of positive psychology was thus collected at baseline to allow for capture of changes post-training.

#### Qualitative Measures

The focus groups’ questions specific to the TTT included participants’ (a) comments on the TTT and suggestions for improvement; (b) personal gain from the TTT; (c) application of the knowledge and skills learned to the intervention, and barriers confronted in the application; and (d) willingness to use the skills learned in future activities.

### Data Analyses

The principle of intention-to-treat (ITT) analysis was adopted. Missing data of participants who withdrew from the study, were lost to follow-up, declined to complete or did not finish the questionnaire, were replaced by their baseline (T1) values. MANOVA analyses were performed initially to detect the time effect on the outcome variables, followed by *post hoc* paired *t*-tests comparing T2, T3, and T4 to baseline scores. A *p* value of less than 0.05 was considered statistically significant. Effect size (Cohen’s *d*) was computed. A positive effect size indicated an increase in the mean score of the outcome, while a negative effect size indicated a decrease. An effect size of 0.2 to <0.5 was considered as small, 0.5 to <0.8 as medium, and 0.8 or above as large (43). Sensitivity analysis was performed by using per protocol (or complete case) analysis, which included participants who completed all assessments (T1–T4) and excluded those with missing data. Results from ITT had the same level of statistical significance and similar effect size as the corresponding per protocol analysis results (Tables S1 and S2 in Supplementary Material). Therefore, to avoid exaggerating the positive effects of the TTT, results from the more conservative ITT method are mainly presented in this paper. All analyses were performed using SPSS 20.0. The qualitative data were analyzed by thematic content analyses, with themes corresponding to three dimensions of the IMTEE. Coding was conducted by two research team members and consensus achieved in the case of discrepancy, to maximize reliability. The quantitative and qualitative results were finally merged to compare, interrelate, and validate results. We used the mixed methods triangulation design (44), which places equal emphasis on quantitative and qualitative data in interpretation.

## Results

### Sociodemographic Characteristics, Previous Experiences, and Role in EFWB

Ninety-three agency staff attended the TTT and 90 completed the baseline survey. Three agency staff declined to participate because of their busy time schedule. Eighty-eight, 63, and 57 agency staff completed the T2, T3, and T4 surveys (follow-up rate: 98%, 70%, and 63%), respectively. The majority were women (80%), aged 25–44 years (71%), had a tertiary degree or above (66%), were registered social service staff (78%), and targeted families in their service (63%). On average, the participants had experience in social service for 10.5 years and been employed in their current organization for 8 years. Their training in positive psychology and research methods prior to the current TTT was perceived as insufficient (4.63 and 3.79 out of 10, respectively). They were involved in the community-based intervention programs of the EFWB Project as program content designers (69%), interventionists (63%), and intervention supporting staff (53%). Some of them had multiple roles in the project (Table 2).

**Table 2:** **Sociodemographic characteristics, previous experiences in relevant trainings, and role in the current project (*N* = 90)**.

	*n* (%) or mean ± SD
Age (years)	
18–24	9 (10)
25–34	40 (44)
35–44	24 (26.7)
≥45	17 (18.9)
Gender	
Male	18 (20)
Female	72 (80)
Education level	
Primary/secondary	3 (3.3)
Non-degree tertiary	28 (31.1)
Degree tertiary or above	59 (65.6)
Registered social service staff	
Yes	70 (77.8)
No	20 (22.2)
Non-governmental organizations’ service target	
Family	57 (63.3)
Children	35 (38.9)
Youth	30 (33.3)
Elderly	16 (17.8)
Mental handicapped	8 (8.9)
Psychically disable	14 (15.6)
Mental rehabilitated	4 (4.4)
New immigrants	15 (16.7)
Ethnic minorities	9 (10)
Others	6 (6.7)
Experience in social service (years)	10.51 ± 7.71
Experience in the current organization (years)	8.01 ± 7.61
Self-reported experiences in having positive psychology training prior to the current train-the-trainer (TTT)^a^	4.63 ± 2.5
Self-reported experiences in having training in research methods prior to the current TTT^a^	3.79 ± 2.48
Role in the Enhancing Family Well-Being Project following TTT (*N* = 63, at 6 months)^b^	
Designed the content of the intervention	48 (68.6)
Interventionist	40 (62.5)
Intervention support staff (not deliver core messages)	31 (52.5)

*^a^Participants were asked to rate the level of sufficiency using a score from 0 to 10 (0 = none, 10 = very sufficient)*.

*^b^This question was asked in the 6-month follow-up survey (T3) on 63 respondents. Most participants played more than one role in the intervention programs*.

Twenty-three agency staff participated in the focus groups. The majority were women (73%), aged 35 years and above (45%), registered social service staff (83%), and had tertiary education (61%). They were mainly responsible for program design (70%) and implementation (83%) in the EFWB Project.

### Reactions to the Training Content and Design

On a scale with a maximum of 5, satisfaction on the contents and design was high, with all scores at T2 being over 3.8. The contents were considered as sufficient (4.18), inspiring (4.03), and applicable (4.15). Participants were satisfied with the design (3.91), brief format (3.80), and time management (3.84), and considered the training had achieved the objectives well (4.10) and increased their knowledge and skills (4.15). Overall scores on the quality, practicability, and satisfaction of the training were about 4.0 (Table 3).

**Table 3:** **Participants’ reactions to the training content and design, assessed immediately after the training (T2) (*N* = 88)**.

Level of satisfaction/overall evaluation	Mean ± SD
The contents were sufficient^a^	4.18 ± 0.58
The contents were inspiring^a^	4.03 ± 0.63
The contents were applicable^a^	4.15 ± 0.52
Design of the training^b^	3.91 ± 0.58
Number of sessions^b^	3.80 ± 0.63
Time management^b^	3.84 ± 0.52
Venue arrangement^b^	3.81 ± 0.77
The training achieved its objectives^a^	4.10 ± 0.55
The training increased my knowledge/skills^a^	4.15 ± 0.54
Overall evaluation: quality of the training^b^	4.03 ± 0.51
Overall evaluation: practicability of the training^b^	4.01 ± 0.56
Overall evaluation: degree of satisfaction^b^	3.97 ± 0.51

*^a^Evaluated on a 5-point Likert scale from strongly disagree (1) to strongly agree (5)*.

*^b^Evaluated on a 5-point Likert scale from very unsatisfactory (1) to excellent (5)*.

The qualitative results corroborated the quantitative finding regarding satisfaction with the TTT. Focus group participants gave positive responses (e.g., “motivating” and “provided a clear direction to the program activities”) to the overall impression of the TTT. Suggestions for improvement were also received from focus groups. Agency staff commented that the training on the research components was not intensive enough and had specific requests for supplementation in specific areas such as proposal writing.

I think the training was very fruitful, as it was impressive … But I needed to digest what I had learned before I could use it with my clients (project support staff, woman, aged 44 years).

The training was good … I liked the training in the Logic Model. It helped to link the behavior indicators with the activity goals. It was useful in our center (project designer and implementer, woman, aged 26 years).

Training from the clinical psychologist was the most useful part … Training from the academic in using the chart (the Logic Model) was essential, because it (the application of the Logic Model in program planning) was different from the simple planning we usually did. I think it would be better to have more training about this model (project implementer, woman, aged 35 years).

I agree that we should learn positive psychology in more depth. Our (past) training in positive psychology has been a bit superficial … The agency staff were the ones implementing the project; it was thus better to provide them with more relevant training and different options (of the themes) to choose from (project implementer, woman, aged 35 years).

We had difficulties writing the project proposal initially. Therefore, a potential way to improve the workshop is by providing more training on proposal writing (project designer and implementer, man, aged 23 years).

### Changes in the Learners

MANOVA showed a significant effect of time on knowledge, self-efficacy, and attitudes in relation to the training contents. Significant increases in knowledge of self-efficacy and attitudes toward the training contents (positive psychology, the Logic Model, process evaluation, and RCT) were found immediately after the training (all *p* < 0.05), with effect sizes for the changes from 0.24 (small) to 1.31 (large) (Table 4). At T3 and T4, the positive effects (increases over baseline) for knowledge of all training contents were sustained (with small to medium effect). Positive changes in attitudes did not persist (*p* > 0.05), except for the item “the Logic Model can provide direction for program design” (T3: Cohen’s *d* = 0.27, *p* = 0.01; T4: Cohen’s *d* = 0.23, *p* = 0.04). For the increases of self-efficacy, variables related to the application of positive psychology and the Logic Model persisted at T3 and/or T4 (*p* < 0.05), while that of the application of process evaluation did not persist (*p* > 0.05) (Table 4). Per protocol analysis showed similar findings (Table S1 in Supplementary Material), including significance level and effect size as those from ITT.

**Table 4:** **Changes in learners’ knowledge of, self-efficacy to use, and attitudes toward the training contents, intention-to-treat analysis (*N* = 90)**.

	T1	T2	T3	T4	T2 vs T1		T3 vs T1		T4 vs T1	

	Mean ± SD	Mean ± SD	Mean ± SD	Mean ± SD	ES	*p*	ES	*p*	ES	*p*
**Positive psychology**										
Knowledge: I know how to apply positive psychology in designing a program	4.04 ± 0.90	4.96 ± 0.56	4.46 ± 0.78	4.32 ± 0.92	1.06	**<0.001**	0.46	**<0.001**	0.36	**0.001**
Self-efficacy: I am competent to use positive psychology as a basis for program design	4.41 ± 0.82	4.99 ± 0.63	4.61 ± 0.73	4.59 ± 0.82	0.84	**<0.001**	0.22	**0.04**	0.20	0.06
Attitude: positive psychology can provide direction for program design	4.54 ± 0.67	4.96 ± 0.50	4.69 ± 0.65	4.62 ± 0.74	0.61	**<0.001**	0.24	**0.03**	0.12	0.28
Attitude: it is worthwhile to use positive psychology to develop programs in the Enhancing Family Well-Being (EFWB) Project	4.80 ± 0.71	5.18 ± 0.57	4.89 ± 0.63	4.81 ± 0.70	0.56	**<0.001**	0.13	0.22	0.02	0.87
Attitude: positive psychology is an ideal way to promote family health, happiness, and harmony	4.89 ± 0.71	5.06 ± 0.55	4.88 ± 0.78	4.78 ± 0.82	0.24	**0.03**	−0.01	0.90	−0.15	0.17
Attitude: positive psychology is an ideal way to promote family relationships	4.82 ± 0.71	5.08 ± 0.67	4.80 ± 0.74	4.76 ± 0.84	0.37	**0.001**	−0.03	0.78	−0.09	0.42
**The Logic Model**										
Knowledge: I know how to apply the Logic Model in planning a program	3.65 ± 1.02	4.87 ± 0.58	4.12 ± 0.93	4.04 ± 0.90	1.22	**<0.001**	0.47	**<0.001**	0.44	**<0.001**
Self-efficacy: I am competent to use the Logic Model as a basis for program planning	3.82 ± 0.98	4.82 ± 0.73	4.13 ± 0.91	4.02 ± 0.90	1.02	**<0.001**	0.34	**0.002**	0.23	**0.03**
Attitude: the Logic Model can provide direction for program design	4.04 ± 0.85	4.89 ± 0.68	4.26 ± 0.87	4.21 ± 0.85	0.90	**<0.001**	0.27	**0.01**	0.23	**0.04**
Attitude: it is worthwhile to use the Logic Model to develop programs	4.12 ± 0.90	4.86 ± 0.70	4.30 ± 0.88	4.27 ± 0.87	0.83	**<0.001**	0.20	0.06	0.18	0.91
Attitude: the Logic Model is an ideal way for program planning	3.97 ± 0.86	4.70 ± 0.79	4.14 ± 0.88	4.13 ± 0.88	0.87	**<0.001**	0.21	**0.045**	0.24	**0.03**
**Process evaluation**										
Knowledge: I know what a process evaluation is	3.84 ± 0.87	4.80 ± 0.56	4.31 ± 0.86	4.18 ± 0.83	1.09	**<0.001**	0.51	**<0.001**	0.45	**<0.001**
Knowledge: I understand the details necessary to conduct a process evaluation	3.64 ± 0.92	4.72 ± 0.67	4.24 ± 0.89	4.06 ± 0.94	1.31	**<0.001**	0.63	**<0.001**	0.48	**<0.001**
Self-efficacy: I can effectively conduct a process evaluation	4.21 ± 0.84	4.66 ± 0.67	4.29 ± 0.75	4.28 ± 0.72	0.52	**<0.001**	0.09	0.39	0.08	0.43
Attitude: process evaluation can provide scientific evidence on the effectiveness of the EFWB Project interventions	4.28 ± 0.77	4.80 ± 0.67	4.41 ± 0.82	4.40 ± 0.78	0.59	**<0.001**	0.15	0.15	0.15	0.15
**Randomized controlled trial**										
Knowledge: I know what an RCT is	3.42 ± 1.18	4.51 ± 0.66	3.87 ± 1.02	3.87 ± 1.08	0.96	**<0.001**	0.38	**<0.001**	0.44	**<0.001**
Knowledge: I know how to conduct an RCT to evaluate the effectiveness of an intervention	3.34 ± 1.08	4.52 ± 0.74	3.72 ± 0.99	3.69 ± 1.01	1.10	**<0.001**	0.34	**0.002**	0.35	**0.001**
Attitude: RCT is a scientific and reliable way to evaluate the effectiveness of an intervention	3.69 ± 1.04	4.59 ± 0.71	3.92 ± 1.01	3.99 ± 0.85	0.86	**<0.001**	0.21	0.05	0.33	**0.003**

*Numbers in bold indicate p < 0.05*.

*The table presents mean score of each item on a 6-point Likert scale from strongly disagree (1) to strongly agree (6)*.

*T1: pre-training survey, T2: immediately post-training survey, T3: 6-month follow-up survey, T4: 12-month follow-up survey*.

*Pair sample *t*-test, *p* value for the difference between baseline (T1) and post-training evaluations (T2, T3, and T4)*.

*ES = effect size (Cohen’s *d*): small = 0.20 to <0.50, medium = 0.50 to <0.80, and large = 0.80 and above*.

The focus group participants indicated enhanced knowledge of positive psychology after the TTT. Some agency staff learned to motivate their clients by improving the format and style of conducting community programs. Improvement in knowledge and skills in positive psychology appeared to be the major changes in learners as indicated by both quantitative and qualitative methods.

I had a better understanding of positive psychology after the training. Our organization has many programs to promote family relationships and this workshop gave us ideas to use in those activities (project implementer, woman, aged 35 years).

There were not just talks (in the TTT). It (the training) was delivered in various formats, including music and video. When we did our own intervention, we also remembered to use a variety of formats, in order to motivate and retain our clients (project designer and implementer, woman, aged 33 years).

### Organization Payoffs

Table 5 shows significant increases in applying positive psychology in program design (Cohen’s *d* = 0.36), and encouraging clients to use positive psychology strategies to improve family relationships and well-being (family health, happiness, and health) in the EFWB Project (Cohen’s *d* ranged from 0.29 to 0.37) at T3 (all *p* < 0.05). These differences did not persist at T4 (all *p* > 0.05). For the application of these concepts beyond the EFWB Project, there were no increases and even some decreases at T3 and T4, except the increase in “encouraging clients to use positive psychology to improve family relationships” at T3 (Cohen’s *d* = 0.24, *p* = 0.03). Per protocol analysis showed similar findings as but slightly greater effect size (Table S2 in Supplementary Material) than the ITT results presented here. For the application of the Logic Model and process evaluation, scores for the EFWB Project were above 3 out of 5, and scores for beyond the EFWB Project were below 3 (Table 5). As described by Zhou et al. (36), after the TTT, the agency staff in the current study had successfully designed and implemented 29 community-based intervention programs to 1,586 clients. The interventions were effective in enhancing participants’ family 3Hs and family relationships.

**Table 5:** **Transfer performance: application of the learning obtained within and beyond the Enhancing Family Well-Being (EFWB) Project, intention-to-treat analysis (*N* = 90)**.

	T1	T3	T4	T3 vs T1		T4 vs T1	
		
	Mean ± SD	Mean ± SD	Mean ± SD	ES	*p*	ES	*p*
**In the past 6 months, how often did you practise the following in the EFWB Project**							
Apply positive psychology in program design	3.37 ± 1.06	3.77 ± 0.91	3.47 ± 1.05	0.36	**0.001**	0.08	0.43
Encourage your clients to use positive psychology to improve family relationships	3.16 ± 1.11	3.49 ± 0.93	3.19 ± 1.03	0.29	**0.007**	0.03	0.76
Encourage your clients to use positive psychology to improve family health	3.07 ± 1.13	3.47 ± 0.97	3.08 ± 1.03	0.37	**0.001**	0.01	0.92
Encourage your clients to use positive psychology to improve family happiness	3.18 ± 1.14	3.50 ± 0.92	3.20 ± 1.03	0.29	**0.007**	0.02	0.83
Encourage your clients to use positive psychology to improve family harmony	3.14 ± 1.11	3.47 ± 0.94	3.19 ± 1.06	0.31	**0.004**	0.06	0.58
Apply the Logic Model in program planning^a^	NA	3.41 ± 0.93	3.07 ± 1.12	NA	NA	NA	NA
Conduct a detailed process evaluation^a^	NA	3.63 ± 0.89	3.25 ± 1.07	NA	NA	NA	NA
**In the past 6 months, how often did you practise the following beyond the EFWB Project**							
Apply positive psychology in designing a program	3.37 ± 1.06	3.44 ± 0.90	3.12 ± 0.93	0.07	0.51	−0.24	**0.02**
Encourage your clients to use positive psychology to improve family relationships	3.16 ± 1.11	3.40 ± 0.96	3.09 ± 1.00	0.24	**0.03**	−0.07	0.51
Encourage your clients to use positive psychology to improve family health	3.07 ± 1.13	3.28 ± 0.97	3.06 ± 1.02	0.20	0.06	−0.01	0.91
Encourage your clients to use positive psychology to improve family happiness	3.18 ± 1.14	3.32 ± 0.96	3.11 ± 1.03	0.14	0.20	−0.07	0.51
Encourage your clients to use positive psychology to improve family harmony	3.14 ± 1.11	3.29 ± 0.96	3.11 ± 1.05	0.15	0.16	−0.03	0.80
Apply the Logic Model in planning a program^a^	NA	2.84 ± 1.12	2.47 ± 1.02	NA	NA	NA	NA
Conduct a detailed process evaluation^a^	NA	2.94 ± 1.11	2.47 ± 1.00	NA	NA	NA	NA

*Numbers in bold indicate p < 0.05*.

*The table presents mean score of each item, on a 5-point Likert scale from never (1) to most of the time (5) of the practice in the past 6 months. T1: pre-training survey, T3: 6-month follow-up survey, T4: 12-month follow-up survey. NA: not available*.

*^a^Baseline data were not available in this item. Mean values were calculated among available cases. N = 63 at T3 and N = 57 at T4*.

*Pair sample *t*-test, *p* value for the difference between baseline (T1) and follow-up evaluations (T3 and T4)*.

*ES = effect size (Cohen’s *d*): small = 0.20 to <0.50, medium = 0.50 to <0.80, and large = 0.80 and above*.

Medium levels of intention to apply the skills learned to other projects were found at T3 (scores ranged from 4.1 to 4.7, out of 6) and at T4 (score ranged from 4.0 to 4.8, out of 6). Over 70% (89.7% at T3 and 73.7% at T4), about 50% (51.7% at T3 and 48.2% at T4), and over 55% (55.7% at T3 and 59.6% at T4) of the participants intended to teach their colleagues positive psychology, the Logic Model, and process evaluation, respectively (Table 6).

**Table 6:** **Intentions to utilize the knowledge and skills in other projects and teach them to colleagues**.

	T3 (*N* = 63)	T4 (*N* = 57)
**I intend to do the following in other projects^a^**	**Mean ± SD**	**Mean ± SD**
Apply positive psychology in program design	4.71 ± 0.77	4.75 ± 0.71
Use the Logic Model in program planning	4.17 ± 0.89	4.00 ± 1.01
Conduct a process evaluation	4.14 ± 0.91	4.14 ± 0.88
**Will you teach the following knowledge and skills to your colleagues?^b^ (percent indicating yes)**	*****n*** (%)**	*****n*** (%)**
The application of positive psychology in program design	52 (89.7)	42 (73.7)
The use of the Logic Model in program planning	31 (51.7)	27 (48.2)
Conducting a process evaluation	34 (55.7)	34 (59.6)

*^a^Measured on a 6-point Likert scale from strongly disagree (1) to strongly agree (6)*.

^b^Participants were asked to choose from the options of “Yes” and “No.”

*T3: 6-month follow-up survey, T4: 12-month follow-up survey*.

Corroborating the above quantitative results, most of the focus group participants reported that they had used the intervention and evaluation skills taught and would use them in future projects and recommend them to others. In the focus groups, those who did not intend to further use the strategies they had been taught in routine practice considered them too time consuming for routine activities. Integrating the quantitative and qualitative findings, the agency staff and community agencies’ enhanced capacity to use positive psychology would be the most significant payoffs for the TTT, since research skills were not considered suitable for agencies’ routine service.

We learned relevant knowledge and skills (from the TTT), and used them to serve our clients. In fact this was a very good learning process. We shall teach what we learned to our colleagues because I think the information is applicable not only to our project, but also to other activities conducted by my colleagues (project designer and implementer, woman, aged 33 years).

I will be more likely to use positive psychology than other skills taught (in future activities) (project designer and implementer, woman, aged 30 years).

They (the training contents) will probably not be used in our future activities, because the application (of the strategies we learned) is time and money consuming. The Logic Model could be used when we apply for some external funding … But in our usual activities, there is not adequate time for us to use the Logic Model in program planning (project supervisor, designer and implementer, woman, aged 31 years).

## Discussion

An integration of our quantitative and qualitative findings suggest that social service agency staff in our study indicated a high level of satisfaction with the training content and design of our brief TTT. The training program immediately and effectively enhanced agency staff’s knowledge, self-efficacy, and positive attitudes toward the content. Enhancement of knowledge of and self-efficacy in using the concepts from the training contents persisted at 6 and for some measures 12 months post-training. Agency staff indicated that they used the skills they had learned in the interventions they developed in the EFWB Project. Application of positive psychology strategies in future community agency-based programs was the major organization payoffs of the current TTT.

Our findings are consistent with that of our previous HFK project (7), suggesting that TTT models are suitable for our large-scale community-based positive psychology intervention projects. The effect sizes for enhancing competence and performance in positive psychology are smaller in the current TTT, in comparison to that of the HFK. This difference is possibly because the earlier TTT focused primarily on positive psychology and offered more intensive training in this limited area. In contrast, the current TTT was shorter, and the content was expanded to include research planning and evaluation skills. Nevertheless, the current TTT resulted in small to medium effects many of which were maintained over a period of 12 months, and the training reached more staff from more community agencies than the earlier project.

Our TTT was beneficial to the enhanced sustainability of the community-based intervention in the EFWB Project. Significant increases in knowledge and self-efficacy in the follow-ups over baseline in our study suggested the persistence of the TTT effects. Lai et al. (7) in the HFK Project also reported similar trends regarding to the changes of agency staff’s knowledge, self-efficacy, and attitudes. Keeping in mind that the goal was not to train researchers, but rather to familiarize front-line mental health staff with the concepts and strategies used by researchers, even a small increase in positive attitudes toward research would enhance the acceptability of future collaborations and interventions in the community. The success and positive impact of the subsequent interventions conducted by the agency staff (36) was important corroboration of the positive effects of the TTT. In addition, the findings on transfer performance implied that the agency staff might be more likely to use and teach positive psychology to their colleagues, than the research skills they acquired. These findings are not surprising as community practitioners are typically more service oriented, interacting with individuals routinely, rather than research or program oriented. Therefore, enhancing the acceptability of the research methods in our TTT could be an area for future improvement. Involving community partners to teach and share their experience in the application of the research methods might be useful to improve the acceptability.

Although many agency staff found the skills were useful in designing projects that were not associated with the current study, some agency staff suggested that the strategies were too demanding in terms of time and resources. Nevertheless, both quantitative and qualitative results demonstrated agency staff’s intention to utilize the skills learned in the TTT in the future, and to share them with their colleagues. It was also possible that our agency staff were occupied in the EFWB Project during the follow-up period and so had little chance to develop or deliver other interventions. A longer period (a few years) of evaluation might better assess the sustainability and generalizability of the training.

This study had several strengths. Our TTT was short; others published in the literature have been more intensive (from 3 to 15 days) (41, 42, 45, 46). Brief and effective training programs have been less frequently reported in the literature [exceptions are Keating et al. (27) and Lucio et al. (28)]. Publication bias could be a problem for short TTT programs with minimal effects, making it difficult to discover cost to benefit information. A unique characteristic of the current program was that it took into account that the staff were from different NGOs serving very different clients. Given that TTT sessions consume resources that are limited, the ability to train individuals in frameworks that are applicable to different populations was a strength of our program. The training was developed and evaluated in an academic and community partnership, which should be more relevant and acceptable. Moreover, the use of both quantitative and qualitative methods in training evaluation is relatively rare in the literature. Most reports on TTTs only provide descriptive and general information about the theoretical framework, development, and design (16, 47–49). Even when TTT evaluations are conducted, they are limited to assessment immediately post-training (20) or compared with the pretraining assessment (18, 50, 51). Our TTT evaluation integrated qualitative and qualitative findings in a triangulation fashion, in order to enrich our understanding of the response. Finally, to avoid exaggerating the positive effects of the training, a conservative ITT method was employed to handle the missing values in the longitudinal data. Similar results obtained from ITT and per protocol analysis implied that participants in the post-training assessments did not exaggerate their positive changes, and social desirability was thus not substantial in the study.

We acknowledge some limitations of the study. The absence of a control group limited the conclusions about whether the changes were specific to the training program. A more rigorous trial could compare the effects of the TTT with a control group using, for example, written materials and handouts alone. Moreover, validated questionnaires were not available in the literature. We developed ours to assess changes in agency staff, by measuring perceptions but not actual knowledge and skills. However, the perceptions that could be influenced by individual’s personality and numerous factors at the time of completing the questionnaire may under- or overestimate the actual knowledge and skills acquired (52).

## Conclusion

This short TTT was built upon a successful training model for community-based interventions implemented in our program of research (7) and modified by reducing the length of the training, increasing the numbers of the agency staff, incorporating training in research methods to maximize and sustain the capacity of the community and the thereby also of the community-based interventions, and including more quantitative variables and qualitative interviews to capture the effects of the training. The findings showed that the TTT expanded capacity among social service agency staff. The training and evaluation could serve as an example for future capacity building among agency staff in large-scale, low cost, and preventive interventions in non-western cultures.

## Author Contributions

Conceived and designed the study: SC, QZ, and THL. Conducted the training: SC, THL, QZ, CL, and AW. Analyzed the data: QZ. Wrote the paper: QZ, SS, THL, and AL. Approved the final version of the manuscript: QZ, SS, THL, SC, AW, CL, and AL.

## Conflict of Interest Statement

The authors declare that the research was conducted in the absence of any commercial or financial relationships that could be construed as a potential conflict of interest. The reviewer CM and handling Editor declared their shared affiliation, and the handling Editor states that the process nevertheless met the standards of a fair and objective review.
