# Supplementary material for: Development and Evaluation of a Train-the-Trainer Workshop for Hong Kong Community Social Service Agency Staff
Source: Front Public Health. 2017 Feb 13;5:15. doi: 10.3389/fpubh.2017.00015 (PMC5303710; doi:10.3389/fpubh.2017.00015)
Supplement: Supplementary file 1 [file table_1.pdf]

Supplementary Table 1. Changes in learners' knowledge of, self-efficacy to use, and attitudes towards the training contents, per-protocol analysis (n=48).

	T1	T2	T3	T4	T2vsT1		T3vsT1		T4vsT1	
	Mean±SD	Mean±SD	Mean±SD	Mean±SD	ES	*p*	ES	*p*	ES	*p*
Positive psychology										
**Knowledge:** I know how to apply positive psychology in designing a program.	4.19±0.84	5.00±0.58	4.71±0.71	4.63±0.79	1.03	**<0.001**	0.54	**0.001**	0.43	**0.004**
**Self-efficacy:** I am competent to use positive psychology as a basis for program design.	4.35±0.89	4.98±0.76	4.73±0.68	4.65±0.86	0.89	**<0.001**	0.37	**0.02**	0.26	0.08
**Attitude:** Positive psychology can provide direction for program design.	4.60±0.68	4.98±0.57	4.77±0.63	4.75±0.76	0.59	**<0.001**	0.26	0.07	0.17	0.24
**Attitude:** It is worthwhile to use positive psychology to develop programs in the EFWB Project.	4.81±0.76	5.31±0.59	4.90±0.66	4.81±0.76	0.73	**<0.001**	0.12	0.40	0.00	1.00
**Attitude:** Positive psychology is an ideal way to promote family health, happiness and harmony.	4.90±0.72	5.06±0.60	4.83±0.81	4.65±0.89	0.21	0.15	-0.07	0.63	-0.26	0.08
**Attitude:** Positive psychology is an ideal way to promote family relationships.	4.85±0.71	5.13±0.71	4.81±0.76	4.69±0.93	0.32	**0.03**	-0.05	0.74	-0.17	0.25
The Logic Model										
**Knowledge:** I know how to apply the Logic Model in planning a program.	3.60±1.11	4.81±0.61	4.33±0.91	4.23±0.81	1.17	**<0.001**	0.60	**<0.001**	0.58	**<0.001**
**Self-efficacy:** I am competent to use the Logic Model as a basis for program planning.	3.69±1.06	4.73±0.89	4.19±0.94	4.02±0.93	0.99	**<0.001**	0.47	**0.002**	0.29	**0.048**
**Attitude:** The Logic Model can provide direction for program design.	3.98±0.89	4.83±0.75	4.33±0.93	4.19±0.92	0.88	**<0.001**	0.34	**0.02**	0.22	0.14
**Attitude:** It is worthwhile to use the Logic Model to develop programs.	4.00±0.99	4.83±0.81	4.31±0.95	4.19±0.96	0.92	**<0.001**	0.29	0.05	0.18	0.23

**Attitude:** The Logic Model is an ideal way for program planning.	3.83±0.91	4.63±0.91	4.10±0.93	4.10±0.95	0.91	**<0.001**	0.28	0.06	0.30	**0.04**
Process evaluation										
**Knowledge:** I know what a process evaluation is.	3.90±0.81	4.79±0.62	4.58±0.68	4.48±0.55	1.08	**<0.001**	0.64	**<0.001**	0.63	**<0.001**
**Knowledge:** I understand the details necessary to conduct a process evaluation.	3.71±0.87	4.73±0.74	4.56±0.68	4.45±0.72	1.30	**<0.001**	0.80	**<0.001**	0.70	**<0.001**
**Self-efficacy:** I can effectively conduct a process evaluation.	4.25±0.91	4.65±0.73	4.40±0.76	4.36±0.71	0.48	**0.002**	0.13	0.38	0.08	0.60
**Attitude:** Process evaluation can provide scientific evidence on the effectiveness of the EFWB Project interventions.	4.19±0.79	4.79±0.74	4.44±0.90	4.38±0.87	0.63	**<0.001**	0.22	0.14	0.17	0.24
Randomized controlled trial (RCT)										
**Knowledge:** I know what an RCT is.	3.33±1.16	4.50±0.68	4.00±0.85	4.00±1.03	1.04	**<0.001**	0.52	**0.001**	0.54	**<0.001**
**Knowledge:** I know how to conduct an RCT to evaluate the effectiveness of an intervention.	3.25±1.10	4.46±0.77	3.85±0.93	3.79±0.97	1.15	**<0.001**	0.47	**0.003**	0.44	**0.004**
**Attitude:** RCT is a scientific and reliable way to evaluate the effectiveness of an intervention.	3.50±1.01	4.52±0.77	3.98±0.84	3.94±0.86	0.96	**<0.001**	0.37	**0.01**	0.38	**0.01**

The table presents mean score of each item on a 6-point Likert scale from strongly disagree (1) to strongly agree (6).
T1: pre-training survey, T2: immediately post-training survey, T3: six months follow-up survey, T4: 12 months follow-up survey
Pair sample t test, *p* value for the difference between baseline (T1) and post-training evaluations (T2, T3, and T4).
ES=effect size (Cohen's d): small=0.10, medium=0.50 and large=0.80.
