# Supplementary material for: Development and Evaluation of a Train-the-Trainer Workshop for Hong Kong Community Social Service Agency Staff
Source: Front Public Health. 2017 Feb 13;5:15. doi: 10.3389/fpubh.2017.00015 (PMC5303710; doi:10.3389/fpubh.2017.00015)
Supplement: Supplementary file 2 [file table_2.pdf]

Supplementary Table 2. Transfer performance: application of the learning obtained within and beyond the EFWB Project, per-protocol analysis (n=48).

	T1	T3	T4	T3vsT1		T4vsT1	
	Mean±SD	Mean±SD	Mean±SD	ES	*p*	ES	*p*
In the past six months, how often did you practise the following in the EFWB Project							
Apply positive psychology in program design	3.31±1.13	3.94±0.86	3.69±1.06	0.47	**0.002**	0.26	0.08
Encourage your clients to use positive psychology to improve family relationships	3.13±1.21	3.63±0.91	3.33±1.01	0.35	**0.02**	0.16	0.26
Encourage your clients to use positive psychology to improve family health	3.08±1.24	3.67±0.97	3.21±1.03	0.45	**0.003**	0.09	0.52
Encourage your clients to use positive psychology to improve family happiness	3.21±1.24	3.67±0.88	3.33±1.02	0.34	**0.02**	0.10	0.48
Encourage your clients to use positive psychology to improve family harmony	3.17±1.19	3.60±0.92	3.35±1.04	0.33	**0.03**	0.16	0.27
Apply the Logic Model in program planning	NA	3.48±0.92	3.10±1.12	NA	NA	NA	NA
Conduct a detailed process evaluation	NA	3.71±0.92	3.31±1.06	NA	NA	NA	NA
In the past six months, how often did you practise the following beyond the EFWB Project							
Apply positive psychology in designing a program	3.31±1.13	3.36±0.92	3.09±0.91	0.05	0.72	-0.21	0.17
Encourage your clients to use positive psychology to improve family relationships	3.13±1.21	3.45±1.00	3.17±0.96	0.25	0.10	0.00	1.00
Encourage your clients to use positive psychology to improve family health	3.08±1.24	3.34±1.05	3.19±0.97	0.20	0.17	0.06	0.71
Encourage your clients to use positive psychology to improve family happiness	3.21±1.24	3.36±1.01	3.21±1.00	0.11	0.45	-0.04	0.81
Encourage your clients to use positive psychology to improve family harmony	3.17±1.19	3.30±1.02	3.23±1.03	0.10	0.50	0.02	0.90
Apply the Logic Model in planning a program	NA	2.81±1.15	2.45±1.02	NA	NA	NA	NA
Conduct a detailed process evaluation	NA	2.87±1.19	2.47±1.02	NA	NA	NA	NA

The table presents mean score of each item, on a 5-point Likert scale from never (1) to most of the time (5) of the practice in the past six months.
T1: pre-training survey, T3: six months follow-up survey, T4: 12 months follow-up survey
Pair sample t test, *p* value for the difference between baseline (T1) and follow-up evaluations (T3, and T4).
ES=effect size (Cohen's d): small=0.10, medium=0.50 and large=0.80. NA: not available
